# Assessment of Patient Preferences for Telehealth in Post–COVID-19 Pandemic Health Care

**DOI:** 10.1001/jamanetworkopen.2021.36405

**Published:** 2021-12-01

**Authors:** Zachary S. Predmore, Elizabeth Roth, Joshua Breslau, Shira H. Fischer, Lori Uscher-Pines

**Affiliations:** 1 RAND Corporation, Boston, Massachusetts; 2 RAND Corporation, Santa Monica, California; 3 RAND Corporation, Pittsburgh, Pennsylvania; 4 RAND Corporation, Arlington, Virginia

## Abstract

**Importance:**

Telehealth use greatly increased in 2020 during the first year of the COVID-19 pandemic. Patient preferences for telehealth or in-person care are an important factor in defining the role of telehealth in the postpandemic world.

**Objective:**

To ascertain patient preferences for video visits after the ongoing COVID-19 public health emergency and to identify patient perceptions of the value of video visits and the role of out-of-pocket cost in changing patient preference for each visit modality.

**Design, Setting, and Participants:**

This survey study was conducted using a nationally representative sample of adult members of the RAND American Life Panel. The data were obtained from the American Life Panel Omnibus Survey, which was fielded between March 8 and 19, 2021.

**Main Outcomes and Measures:**

Preferences for video visits vs in-person care were analyzed in the survey. The first question was about participants’ baseline preference for an in-person or a video visit for a nonemergency health issue. The second question entailed choosing between the preferred visit modality with a cost of $30 and another modality with a cost of $10. Questions also involved demographic characteristics, experience with video visits, willingness to use video visits, and preferences for the amount of telehealth use after the COVID-19 pandemic.

**Results:**

A total of 2080 of 3391 sampled panel members completed the survey (participation rate, 61.3%). Participants in the weighted sample had a mean (SE) age of 51.1 (0.67) years and were primarily women (1079 [51.9%]). Most participants (66.5%) preferred at least some video visits in the future, but when faced with a choice between an in-person or a video visit for a health care encounter that could be conducted either way, more than half of respondents (53.0%) preferred an in-person visit. Among those who initially preferred an in-person visit when out-of-pocket costs were not a factor, 49.8% still preferred in-person care and 23.5% switched to a video visit when confronted with higher relative costs for in-person care. In contrast, among those who initially preferred a video visit, only 18.9% still preferred a video visit and 61.7% switched to in-person visit when confronted with higher relative costs for video visits.

**Conclusions and Relevance:**

This survey study found that participants were generally willing to use video visits but preferred in-person care, and those who preferred video visits were more sensitive to paying out-of-pocket cost. These results suggest that understanding patient preferences will help identify telehealth’s role in future health care delivery.

## Key Points

**Question:**

What role do US adults envision for telehealth in their future medical care?

**Findings:**

In this survey study of 2080 adults, most respondents were willing to use video visits in the future but, when presented with the choice between an in-person or a video visit for nonemergency care, most preferred in-person care. Willingness to pay for preferred visit modality was higher for those who preferred in-person care, and those who preferred video visits were more sensitive to out-of-pocket cost.

**Meaning:**

The findings of this study suggest that awareness of patient preferences will help define telehealth’s role in US health care after the COVID-19 pandemic.

## Teaser

This survey study assesses the factors associated with continued use of telehealth services, including experience with, cost of, and benefits or barriers to video-based clinician visits among adult patients.

## Introduction

Telehealth use rapidly increased in the US during the COVID-19 pandemic, with many health care practitioners offering telephone or video visits to reduce the potential for virus spread.^1^ It is unclear how telehealth will be used within the US health care system after the pandemic, with early evidence suggesting that telehealth use is decreasing as patients and clinicians resume in-person care.^2^

Payers are currently making decisions about postpandemic reimbursement policy and considering telehealth’s implications for quality, costs, fraud, and abuse. These decisions are informed by claims data analyses of telehealth utilization^3^ and clinician surveys and interviews regarding their experiences with delivering telemedicine.^4,5^ Surveys of the general public have been conducted, but they generally describe the different visit modalities available and the interest or willingness to use telehealth. A nationally representative survey from 2020 found that 40% of patients with a health condition used telehealth in the spring of 2020.^6^ Surveys from before the COVID-19 pandemic found that 49% to 66% of the respondents were interested in using video visits, with greater preference for telehealth when seeing their own clinician vs a new clinician.^7,8,9,10^ Although numerous studies have shown that patients are satisfied with telehealth and appreciate its convenience,^11,12,13^ data are scarce on the role that patients would like telehealth to play in their overall care, including within hybrid care models, and the perceived value of telehealth compared with other visit modalities. Such information is key to understanding the implications of different coverage and reimbursement policies, including cost sharing, for the demand for telehealth and the potential backlash of rolling back the telehealth flexibilities that were implemented for the COVID-19 pandemic.

We conducted a nationally representative survey study using conjoint analysis, a technique in which respondents make hypothetical choices between alternatives that vary in 1 or more attributes, to ascertain patient preferences for video visits after the ongoing COVID-19 public health emergency. Specifically, we sought to identify patient perceptions of the value of video visits and the role of out-of-pocket cost in changing patient preferences for each visit modality. We also asked participants about their willingness to use video visits in the future and their preferences for the amount of video visits that they will incorporate in their medical care after the COVID-19 pandemic.

## Methods

We conducted this survey using the RAND American Life Panel, which produces nationally representative estimates from a probability-based representative sample of the US adult population aged 20 years or older.^14^ The survey was approved by the RAND Institutional Review Board, which waived the informed consent requirement because the American Life Panel survey data collection fell under the adult interview and survey exemption. We followed the American Association for Public Opinion Research (AAPOR) reporting guideline.

The American Life Panel recruited its first wave of participants in 2002 and is continually refreshed to create a total current panel size of approximately 6000 participants.^15^ Participants have been recruited from other nationally representative probability-based panels, sampling in zip codes with high percentages of racial minority groups, and random digit dialing.^15^ Panel participants are given internet-connected devices and are paid for completing surveys. For the present survey study, the data were obtained from the American Life Panel Omnibus Survey, which was fielded between March 8 and 19, 2021. Weights are generated through a raking process using the demographic data of participants (sex, age, race and ethnicity, educational level, household income, and number of household members) rather than the national estimates of these variables in the Current Population Survey Annual Social and Economic Supplement.^15^

We hypothesized that some people would prefer in-person care and others would prefer video visits. Thus, we asked respondents first about their baseline preference for an in-person or a video visit for a nonemergency health issue that their personal physician indicated could be handled through either visit modality. Given that telehealth is an umbrella term and can include telephone visits, video visits, store-and-forward encounters, and remote patient monitoring, we narrowed the scope of the questions by specifying video visits.

For those who expressed a preference for either an in-person or a video visit, we posed a second question, offering their preferred modality for an out-of-pocket cost of $30 or the other modality for an out-of-pocket cost of $10, and asking which option they preferred. These costs were selected because they represent the lower and higher bounds of typical co-payments for outpatient visits.^16^ The cost difference of $20 was used to assess the relative value placed on each visit modality. We also asked questions about demographic characteristics, experience with video visits, willingness to use video visits, and preferences for the amount of telehealth use after the COVID-19 pandemic. Participants self-reported their race and ethnicity, choosing from a set of options defined by the American Life Panel survey investigators. For ethnicity, the question was, “Do you consider yourself Hispanic or Latino?” with a yes or no option. For race, the question was to choose from these options: White/Caucasian, Black/African American, Asian or Pacific Islander, American Indian or Alaska Native, or other race. We assessed race and ethnicity to identify potential disparities in access to telehealth and to capture any differences in preferences for future use of telehealth. Visit modality–related questions are shown in the eTable in the Supplement.

### Statistical Analysis

We used the American Life Panel response weights to produce nationally representative estimates of answers to the survey questions.^17^ We calculated descriptive statistics (counts, means, SEs) and cross-tabulated frequencies and percentages. We used Rao-Scott χ^2^ tests for bivariate comparisons of in-person or video visit preferences by demographic variables (sex, age, race and ethnicity, educational level, family income, urbanicity, and previous use of video visits) and for adjusted logistic regression analyses of binary outcomes of telehealth preferences after the COVID-19 pandemic by demographic variables (sex, age, race and ethnicity, educational level, family income, urbanicity, and previous use of video visits). Missing data were limited (<1% of all variables) and likely random; entries with missing values were dropped from the adjusted analyses.

Analyses were conducted using SAS/STAT software, version 9.4 of the SAS System for Linux (SAS Institute). The threshold for statistical significance was a 2-sided *P* < .05.

## Results

Of the 3391 sampled panel members, 2080 completed the survey, for a 61.3% participation rate. Of the final weighted sample 51.9% of participants were women (n = 1079) and 48.1% were men (n = 1001), with a mean (SE) age of 51.1 (0.67) years. Among the participants, 18.2% self-identified as Hispanic/Latino, 11.8% as non-Hispanic Black/African American, and 63.8% as non-Hispanic White/Caucasian. Complete weighted demographic characteristics of the panel are described in Table 1. Only 7 respondents did not answer all of the demographic questions; we included their answers in the descriptive statistics and univariate analyses of the variables for which they provided data, but we excluded all 7 respondents from the multivariate analyses. Forty-five percent of participants reported having 1 or more video visits with health care practitioners since March 2020. Most respondents were willing to use video visits as indicated on a 5-point scale: 61.4% said they were very willing or willing, whereas only 8.5% were unwilling. Of the 45.0% of respondents who had used telehealth since March 2020, only 2.3% reported that they were unwilling to use telehealth in the future.

**Table 1.** Demographic Characteristics of Survey Participants

Characteristic	Unweighted frequency, No.	Weighted % (SE)
Sex		
Female	1162	51.9 (2.0)
Male	918	48.1 (2.0)
Age group, y		
20-39	226	28.7 (2.2)
40-59	692	37.6 (1.9)
≥60	1162	33.8 (1.6)
Race and ethnicity^a^		
Hispanic/Latino	268	18.2 (1.7)
Non-Hispanic		
Black/African American	175	11.8 (1.3)
White/Caucasian	1521	63.8 (2.0)
Other^b^	116	6.2 (1.0)
Educational level		
<High school	54	8.1 (1.4)
High school diploma to associate’s degree	922	54.6 (2.0)
≥Bachelor’s degree	1104	37.3 (1.8)
Family income, $		
<35 000	462	23.7 (1.8)
35 000-59 999	456	20.5 (1.6)
60 000-99 999	540	24.1 (1.6)
≥100 000	619	31.7 (1.9)
Urbanicity		
Rural or small town; population <50 000	460	21.6 (1.7)
Small to midsize or large city; population ≥50 000	1617	78.4 (1.7)
Previous use of telehealth		
Had used telehealth	900	45.0 (2.0)
Had not used telehealth	1180	55.0 (2.0)
Willingness to use video visit in the future		
1 (Unwilling)	183	8.5 (1.1)
2	188	7.6 (0.9)
3	479	22.6 (1.8)
4	460	22.1 (1.7)
5 (Very willing)	769	39.3 (1.9)
Preference for amount of video visits after COVID-19 pandemic		
None: I prefer all of my care to be in person	731	33.5 (1.9)
A little	580	30.1 (1.9)
Some	556	26.1 (1.7)
As much as possible	212	10.3 (1.0)

^a^ Race and ethnicity were self-reported by survey participants, who chose from a set of race and ethnicity options defined by the American Life Panel investigators.

^b^ The Other category included Asian or Pacific Islander, American Indian or Alaska Native, or other race.

When out-of-pocket costs were not a factor in the decision to seek care for a nonemergency health issue, 53.0% of participants preferred an in-person visit, 20.9% preferred a video visit, and 26.2% did not have a preference or did not know. Univariate analyses of preferences are shown in Table 2. Previous use of video visits was significantly associated with preference for video visits in the future. Among participants who had participated in a video visit since March 2020, 44.2% preferred an in-person visit and 31.4% preferred a video visit (*P* < .001). In comparison, of the participants without experience with video visits, 60.2% preferred an in-person visit and only 12.2% preferred a video visit (*P* < .001) (Table 2).

**Table 2.** Preferences for In-Person or Video Visit by Demographic Characteristics

Characteristic	Unweighted frequency, No.	Preference, weighted %				*P* value
		In-person visit	Video visit	None or both	Do not know	
Total sample	2080	53.0	20.9	22.9	3.3	
Sex						
Female	1162	49.1	23.9	23.1	3.9	.16
Male	918	57.2	17.6	22.7	2.6	
Age group, y						
20-39	226	42.3	25.9	29.6	2.2	<.001
40-59	692	50.8	24.4	20.7	4.0	
60 and up	1162	64.5	12.6	19.7	3.3	
Race and ethnicity^a^						
Hispanic/Latino	268	58.6	22.9	15.2	3.3	.02
Non-Hispanic						
Black/African American	175	64.1	16.5	14.2	5.2	
White/Caucasian	1521	49.3	22.0	26.3	2.4	
Other^b^	116	53.7	11.8	26.6	7.9	
Educational level						
<High school	54	64.4	17.8	13.4	4.3	.03
High school diploma to associate’s degree	922	55.6	18.8	21.2	4.4	
≥Bachelor’s degree	1104	46.7	24.5	27.3	1.4	
Family income, $						
<35 000	462	55.1	16.0	21.0	7.9	<.001
35 000-59 999	456	62.7	16.6	19.0	1.8	
60 000-99 999	540	54.5	21.9	21.9	1.7	
≥100 000	619	44.0	26.5	27.5	2.0	
Urbanicity						
Rural or small town; population <50 000	460	55.9	17.6	24.3	2.2	.62
Small to midsize or large city; population ≥50 000	1617	52.2	21.8	22.5	3.5	
Previous use of video visits						
Had used video visits	900	44.2	31.4	22.3	2.1	<.001
Had not used video visits	1180	60.2	12.2	23.4	4.2	

^a^ Race and ethnicity were self-reported by survey participants, who chose from a set of race and ethnicity options defined by the American Life Panel investigators.

^b^ The Other category included Asian or Pacific Islander, American Indian or Alaska Native, or other race.

Younger adults were more likely than older adults to prefer video visits. Among participants aged 20 to 39 years, 42.3% preferred in-person visits and 25.9% preferred video visits compared with 64.5% of participants who preferred in-person visits and 12.6% who preferred video visits in the 60 years or older group (*P* < .001). Individuals with higher income were also more likely than those with lower income to prefer video visits. Among individuals making under $35 000 per year, 55.1% preferred in-person visits and 16.0% preferred video visits (*P* < .001). Meanwhile, of the participants making more than $100 000 per year, 44.0% preferred in-person visits and 26.5% preferred video visits (*P* < .001) (Table 2).

Black/African American respondents were more likely than respondents of other races and ethnicities to prefer in-person care (64.1% vs 51.5%; *P* = .02), and Hispanic/Latino respondents were more likely to prefer video visits compared with individuals from other racial and ethnic groups (22.9% vs 20.4%; *P* = .02). Differences in modality preferences by race and ethnicity were statistically significant. Educational level was also associated with preferences for video visits, with 64.4% of respondents who did not complete high school preferring in-person care compared with 55.6% of high school graduates and 46.7% of college graduates (*P* = .03). Conversely, 24.5% of respondents with a bachelor’s degree or higher preferred video visits compared with 17.8% of those who did not complete high school and 18.8% with a high school diploma (*P* = .03). These differences were statistically significant.

Those who preferred in-person care (weighted n = 1101) when out-of-pocket cost was not considered were asked about their preferences for a more expensive in-person visit ($30) and a less expensive video visit ($10) (weighted counts may not add up to reported totals because of rounding). When faced with this scenario, 548 participants (49.8%) still preferred an in-person visit, 259 (23.5%) switched and preferred a video visit, and 295 (26.8%) did not have a preference or did not know (Table 3).

**Table 3.** Visit Modality Preferences for Nonemergency Health Issues

Survey response	Preference with out-of-pocket cost consideration					
	Weighted No.	No. (%)				
		Preferred modality with a $30 co-pay/cost	Other preferred modality with a $10 co-pay/cost	None or both modalities	Do not know	Missing data
I’d prefer an in-person visit	1101	548 (49.8)	259 (23.5)	171 (15.5)	124 (11.2)	0 (0)
I’d prefer a video visit	434	82 (18.9)	268 (61.7)	70 (16.1)	13 (3.0)	1 (0.2)
I have no preference or equal preference	476	NA	NA	NA	NA	NA
I do not know	68	NA	NA	NA	NA	NA

Abbreviation: NA, not applicable.

Those participants who preferred video visits (weighted n = 434) when out-of-pocket cost was not a factor were asked about their preferences for a more expensive video visit ($30) and a less expensive in-person visit ($10). When faced with this scenario 82 participants (18.9%) still preferred a video visit, 268 (61.7%) switched and preferred an in-person visit, and 83 (19.1%) did not have a preference or did not know (Table 3). For both groups of respondents, no demographic variables were associated with different probabilities of switching visit modalities on the basis of costs.

Using these stated preferences, we explored participants’ willingness to pay for their preferred visit modality for nonemergency visits in the general population (Figure). Of the 2078 participants who completed the questions, 978 (47.0%) were willing to pay for an in-person visit and 548 (26.3%) valued that preference at $20 more than the cost of a video visit. Nearly a quarter of the population (22.9% [n = 476]) valued both visit modalities equally and presumably would opt for whatever visit modality was the least expensive. In addition, 420 respondents (20.2%) were willing to pay for video visits when choosing between an in-person visit and a video visit for a nonemergency health issue, and only 82 respondents (3.9%) valued video visits at $20 more than the cost of an in-person visit. A total of 205 participants (9.9%) stated that they did not know their preference for 1 of the 2 questions.

**Figure.**
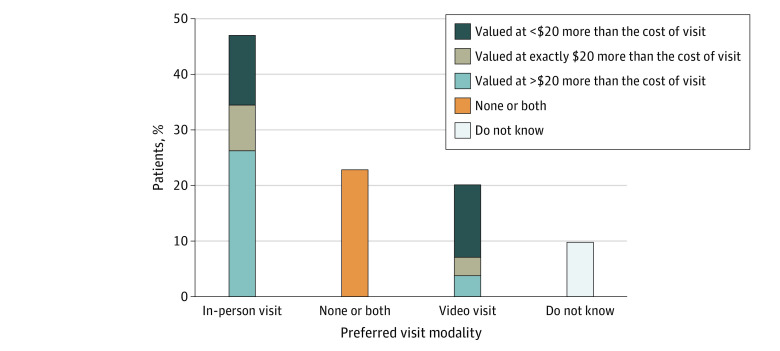
Preferred Visit Modality and Perceived Relative Value (Weighted)

When all participants were asked how much of their medical care they wanted to receive through video visits after the COVID-19 pandemic, 731 (33.5%) preferred all of their care to be in person and 1348 (66.5%) wanted at least some video visits (580 [30.1%] wanted a little, 556 [26.1%] wanted some, and 212 [10.3%] wanted as much as possible) (Table 1). The odds ratios (ORs) from adjusted models are found in Table 4, both with and without previous telehealth use as a covariate. In adjusted models with previous telehealth use included, experience with video visits was associated with greater likelihood of wanting at least some (vs no) video visits (OR, 3.01; 95% CI, 2.13-4.25; *P* < .001), as were younger age (20-39 years: OR, 3.13; 95% CI, 1.70-5.75; *P* < .001) and having a higher family income. Lower family income was associated with lower likelihood of wanting at least some (vs no) video visits (<$35 000: OR, 0.42; 95% CI, 0.25-0.70; *P* < .001).

**Table 4.** Results of Logistic Regression of Preference for Telehealth After the COVID-19 Pandemic (N = 2073)

Variable	Prevalence		Adjusted model			
	% Preferring at least a little telehealth	*P* value	With previous telehealth use		Without previous telehealth use	
			OR (95% CI)	*P* value	OR (95% CI)	*P* value
Sex						
Female	66.8	.84	1.03 (0.72-1.46)	.89	1.12 (0.79-1.58)	.53
Male	66.1		1 [Reference]		1 [Reference]	
Age group, y						
20-39	77.6	<.001	3.13 (1.70-5.75)	<.001	3.24 (1.77-5.95)	<.001
40-59	70.8		2.34 (1.66-3.28)	<.001	2.42 (1.72-3.40)	<.001
≥60	52.2		1 [Reference]		1 [Reference]	
Race and ethnicity^a^						
Hispanic/Latino	64.8	.71	0.77 (0.47-1.29)	.32	0.72 (0.44-1.19)	.20
Non-Hispanic						
Black/African American	61.4		0.70 (0.37-1.33)	.28	0.81 (0.44-1.49)	.49
White/Caucasian	67.5		1 [Reference]		1 [Reference]	
Other^b^	70.0		0.86 (0.42-1.77)	.68	0.78 (0.39-1.55)	.48
Educational level						
<High school	53.4	<.001	0.49 (0.22-1.08)	.08	0.53 (0.23-1.23)	.14
High school to associate’s degree	61.0		0.80 (0.56-1.15)	.22	0.73 (0.52-1.03)	.08
≥Bachelor’s degree	77.3		1 [Reference]		1 [Reference]	
Family income, $						
<35 000	53.2	<.001	0.42 (0.25-0.70)	<.001	0.41 (0.25-0.69)	<.001
35 000-59 999	62.0		0.67 (0.42-1.06)	.09	0.60 (0.38-0.94)	.03
60 000-99 999	67.1		0.74 (0.46-1.21)	.24	0.69 (0.43-1.11)	.13
≥100 000	78.7		1 [Reference]		1 [Reference]	
Urbanicity						
Rural or small town; population <50 000	57.8	.02	0.70 (0.47-1.06)	.09	0.65 (0.42-0.99)	.04
Small to midsize or large city; population ≥50 000	68.8		1 [Reference]		1 [Reference]	
Previous use of video visits						
Had used video visits	79.8	<.001	3.01 (2.13-4.25)	<.001	NA	NA
Had not used video visits	55.6		1 [Reference]		1 [Reference]	

Abbreviations: NA, not applicable; OR, odds ratio.

^a^ Race and ethnicity were self-reported by survey participants, who chose from a set of race and ethnicity options defined by the American Life Panel investigators.

^b^ The Other category included Asian or Pacific Islander, American Indian or Alaska Native, or other race.

## Discussion

In this survey of a nationally representative sample, we found a general willingness to use video visits among US adult respondents. However, when faced with a choice between an in-person or a video visit for a nonemergency health issue, participants generally preferred in-person care, and those who were younger, had higher income, and had a higher educational level were more likely to opt for video visits. Experience with telehealth was associated with preference for video visits, and only 2.3% of those who had participated in a video visit were unwilling to do so again. This finding suggests that, although many participants used telehealth for the first time during the COVID-19 pandemic out of necessity, their experiences were beneficial enough to encourage ongoing use.

Only 33.5% of participants did not see any role for video visits in their medical care. These respondents were generally older, had lower income, lived in more rural areas, and had lower educational level. Although telehealth can expand access to care for underserved populations (eg, those with low family income, with lower educational levels, belonging to racial and ethnic minority groups, and living in rural areas) if deployed in a targeted manner, findings of this survey suggest that these populations may be the least likely to demand it and that ongoing efforts to promote equity of access to telehealth need to consider these preferences.^18^ Further research is needed to explore the reason that certain patients are not interested in hybrid care models, which combine in-person and video visits. For example, it is not clear whether these patients simply do not value telehealth as much as in-person care or do not view telehealth as feasible or practical given their personal circumstances (eg, lack of broadband internet connection and limited digital literacy).^19^

Those who preferred in-person care in the choice task had a higher willingness to pay for their preferred visit modality compared with those who preferred video visits. Those who preferred video visits were more sensitive to out-of-pocket cost than those who preferred in-person visits, as a $20 increase in cost was associated with switching from video visits to in-person care. This difference in perceived value has many potential explanations. Patients may like telehealth in certain circumstances (eg, care for minor acute conditions) but may not perceive video visits to have the same value as in-person care. For example, they may not believe that clinicians are able to offer an equivalent service or are putting in as much clinical effort.^20,21^ Patients may perceive that in-person visits allow for easier referrals for diagnostic testing. Alternatively, some patients may perceive benefits to in-person care that even the highest quality telehealth visit cannot replicate (eg, rapport with clinician as well as ritual and structure of coming in person), outweighing the additional costs associated with in-person care (eg, travel and time costs).

This survey study, to our knowledge, was among the first efforts to explore the value of different visit modalities in the US population and provide important insights into the role that telehealth can play after the COVID-19 pandemic. Although improving the delivery of telehealth is important, it is only part of the story. Awareness of patient preferences will help to identify telehealth’s role in postpandemic health care delivery.

### Limitations

This study has some limitations. First, with the limited conjoint questions that we asked participants, we were unable to conduct a full willingness-to-pay analysis to identify the mean willingness to pay for each visit modality, but we were able to provide some insight into the general value that a nationally representative sample ascribes to each visit modality. Second, the stated preferences approach also had limitations. Respondents may be unfamiliar with making choices between hypothetical options and may have never had to choose between telehealth and in-person care in the past. Third, we were limited in what demographic data were available from the American Life Panel. For example, data on health status were lacking in the American Life Panel Omnibus Survey. Respondents with poor health status may be more or less interested in video visits than those with good health. In addition, respondents may have certain health conditions that make video visits more or less feasible. For example, patients with stable chronic conditions that can be managed through prescription medications, such as diabetes, may be easier to manage than patients with health conditions that require physical examinations, such as cardiac disease. However, lack of such data was not a major limitation given that the goal of this study was to produce nationally representative estimates of preferences. The absence of unmeasured potential confounders did not affect the ability of the study to produce those estimates.

## Conclusions

This survey study found that adult respondents were generally willing to use video visits but preferred in-person care to a video visit for a nonemergency health issue. Previous use of telehealth was associated with preference for video visits, and those who preferred video visits were more sensitive to out-of-pocket cost. Awareness of patient preferences will help to identify telehealth’s role in postpandemic health care delivery.

## Notes

Supplement. **eTable.** Telehealth-Related Survey Questions
