# Supplementary material for: Assessment of Patient Preferences for Telehealth in Post–COVID-19 Pandemic Health Care
Source: JAMA Netw Open. 2021 Dec 1;4(12):e2136405. doi: 10.1001/jamanetworkopen.2021.36405 (PMC8637257; doi:10.1001/jamanetworkopen.2021.36405)
Supplement: Supplement. — eTable. Telehealth-Related Survey Questions [file jamanetwopen-e2136405-s001.pdf]

Supplementary Online Content

Predmore ZS, Roth E, Breslau J, Fischer SH, Uscher-Pines L. Assessment of patient preferences for telehealth in post–COVID-19 pandemic health care. *JAMA Netw Open*. 2021;4(12):e2136405. doi:10.1001/jamanetworkopen.2021.36405

eTable. Telehealth-Related Survey Questions

This supplementary material has been provided by the authors to give readers additional information about their work.

© 2021 Predmore ZS et al. *JAMA Network Open.*

eTable. Telehealth-Related Survey Questions

Question	Answer choices	Who was asked?
Q1. Imagine that after the pandemic you have a non-emergency health issue that your doctor says can be handled in-person in the doctor's office or by video. What would you prefer?	❍ I'd prefer an in-person visit<br>❍ I'd prefer a video visit<br>❍ I have no preference or equal preference<br>❍ I don't know	All respondents
Q2a. Imagine that after the pandemic you have a non-emergency health issue that your doctor says can be handled in-person in the doctor's office or by video. What would you prefer?	❍ An in-person visit with a $30 co-pay/cost to you<br>❍ A video visit with a $10 co-pay/cost to you<br>❍ I have no preference between the above options<br>❍ I don't know	Respondents who preferred an in-person visit in Q1.
Q2b. Imagine that after the pandemic you have a non-emergency health issue that your doctor says can be handled in-person in the doctor's office or by video. What would you prefer?	❍ An in-person visit with a $10 co-pay/cost to you<br>❍ A video visit with a $30 co-pay/cost to you<br>❍ I have no preference between the above options<br>❍ I don't know	Respondents who preferred a video visit in Q1.
Q3. On a 1-5 scale, rate your willingness to participate in a video visit with a doctor for a non-emergency health issue…	❍ 1 Unwilling<br>❍ 2<br>❍ 3<br>❍ 4<br>❍ 5 Very willing	All respondents
Q4. After the pandemic is over, how much of your medical care would you like to have by video rather than in-person?	❍ None: I prefer all of my care to be in-person<br>❍ A little<br>❍ Some<br>❍ As much as possible	All respondents

© 2021 Predmore ZS et al. *JAMA Network Open.*
